# Jar-RetinexNet: A System for Non-Uniform Low-Light Enhancement of Hot Water Heater Tank Inner Walls

**DOI:** 10.3390/s25237121

**Published:** 2025-11-21

**Authors:** Wenxin Cao, Lei Guo, Juanhua Cao, Weijun Wu

**Affiliations:** 1 School of Computer Science and Engineering, University of Electronic Science and Technology of China, Chengdu 611731, China; 202352081237@std.uestc.edu.cn (W.C.); leiguo@uestc.edu.cn (L.G.); 2 School of Automotive and Aviation, Jiangxi Technical College of Manufacturing, Nanchang 330095, China; amyott@163.com; 3 School of Advanced Manufacturing, Nanchang University, Nanchang 330031, China

**Keywords:** electric water heater enamel, hardware-software system, jar-RetinexNet, Heater Tank Inner Wall Dataset

## Abstract

The manual inspection of electric water heater enamel is inefficient and unreliable, a challenge stemming from the tank’s narrow (approx. 50 mm) aperture that creates extremely dim, non-uniform lighting. Existing enhancement algorithms struggle with such complex industrial imagery. To address this, we propose an integrated hardware-software system: the three-axis Image Acquisition Robot (IAR) and Interactive Visualization Enhancement Software (IVES). Using this system, we constructed and released the first Heater Tank Inner Wall (HTIW) dataset, containing 900 real-world images. We further introduce jar-RetinexNet, a Retinex-based network featuring a Feature Preservation Attention Module (FPAM), a Cascaded Channel-Spatial Attention Module (CSAM) for precise decomposition, and a Random Affine Generation (RAG) module for generalization. Experiments show that jar-RetinexNet significantly outperforms state-of-the-art methods, achieving the best no-reference quantitative scores on our HTIW dataset: a BRISQUE of 25.4457 and a CLIPIQA of 0.3160.

## 1. Introduction

**Figure 1:**
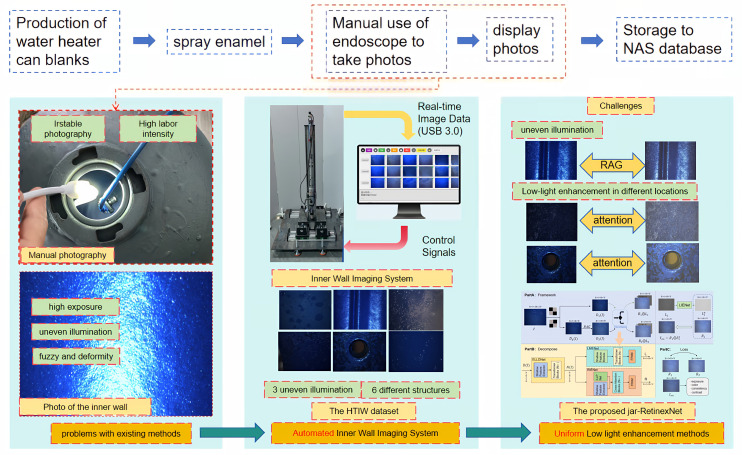
Traditional manual photography challenges in hot water heater tank inner wall inspection and the proposed enhancement system. The blue arrows represent the sequential workflow of the production and inspection process. The red curved arrow in the central panel indicates the control signals sent from the workstation to the imaging hardware.

Electric Storage Water Heaters (ESWH) are ubiquitous household appliances globally, with an average annual production of approximately 50 million units over the past five years [1]. Their safety and durability are of paramount importance for public welfare [2]. Figure 1 illustrates the manufacturing process of ESWH in an industrial setting. To prevent rust and corrosion within the high-temperature and high-humidity water storage environment, the application of a complete enamel coating on the inner tank surface is the industry-standard protective technology [3]. Consequently, acquiring clear images of the inner tank wall is a prerequisite and essential step for quality control in production. In industry, numerous patents have been disclosed for automated visual acquisition systems targeting ESWH inner tanks. Similarly, in academia, various robotic systems have been developed for inspecting analogous internal surfaces, such as penstocks [4]. However, due to the ESWH’s narrow aperture (approx. 50 mm), coupled with a dimly lit and highly curved geometric environment, image acquisition remains exceptionally challenging. Therefore, developing a method for the rapid, comprehensive, and clear acquisition of images from within the ESWH inner tank remains a critical and urgent technical problem in this field.

Existing methods for acquiring images of the ESWH inner tank wall (referred to as VE for Visual Examination) can be broadly categorized into automated visual acquisition systems and manual visual inspection. Automated systems are mechatronic solutions that integrate front-end endoscopic imaging hardware with back-end software control algorithms. Although industrial endoscopes can capture images of the inner wall, their deployment within the narrow aperture necessitates a wide-angle lens design, which results in images that are clear at the center but suffer from peripheral blurring. Furthermore, while some devices have been specifically designed to inspect weld seams on the inner wall, their limited acquisition scope prevents a comprehensive quality assessment of the entire enamel coating. Manual visual inspection, which relies on operators using industrial endoscopes, is intuitive and straightforward. However, as shown in Figure 1, this method is labor-intensive, highly subjective, and prone to producing images with over-exposure, blurred details, and non-uniform illumination. These quality issues severely hamper the accuracy of subsequent analysis by vision algorithms.

To address the challenge of rapid, comprehensive, and clear VE acquisition in ESWH, we have designed a complete enhancement hardware system. This system comprises a three-axis Image Acquisition Robot (IAR) and an Interactive Visualization Enhancement Software (IVES) deployed on an industrial computer. The IAR, through its three axes of motion (longitudinal lifting, central axis rotation, and 180-degree end-effector flipping), drives a high-resolution industrial camera to perform a step-wise scan and capture images of the inner tank wall without dead zones. The acquired images are transmitted in real-time via a high-speed interface to IVES, which not only provides a visual representation of the unwrapped inner tank surface but also performs targeted image enhancement, yielding high-quality data for subsequent analysis. The system is designed to achieve comprehensive, efficient, and visual enhancement of images suffering from Non-uniform Illumination (NUI) and to supply data for downstream defect detection workflows. Field tests at an ESWH factory have demonstrated that our enhancement system effectively accomplishes the NUI enhancement task for VE.

To validate the proposed enhancement system, a dedicated Heater Tank Inner Wall (HTIW) dataset is required to support VE research. However, to the best of our knowledge, no such public dataset is currently available. To fill this gap, we utilized our IAR to collect a large volume of internal VE images from an ESWH factory. Under the guidance of professional quality control personnel, we curated the HTIW dataset from this collection. The dataset contains 900 images, encompassing three typical categories of non-uniform illumination challenges and six representative internal geometric regions (weld seams, mounting holes). To ensure the dataset faithfully reflects real-world industrial challenges, we have preserved the original image resolution without any cropping or preprocessing. We have also identified and analyzed two primary challenges and their three sub-categories present within the HTIW dataset.

Although low-light enhancement methods based on Retinex theory [5] have achieved significant progress [6,7,8], they still face challenges when processing images with non-uniform illumination and complex reflective surfaces, such as those in the HTIW dataset. To enhance the images of water heater inner tanks more effectively, we contend that two core questions must be addressed:

1. How can the adverse effects of non-uniform illumination be eliminated? Non-uniform lighting not only severely degrades the visual quality of images but also significantly impairs the accuracy of downstream task algorithms, such as defect detection.
2. How can adaptive enhancement be performed for different illumination regions? The lighting conditions vary dramatically across different areas within the water heater tank. Without an adaptive approach, enhancement results are highly susceptible to localized over-exposure (over-enhancement) or loss of detail (under-enhancement).

Motivated by these considerations, we propose a physically interpretable non-uniform low-light enhancement network, jar-RetinexNet (shown conceptually in Figure 1, with the detailed end-to-end framework presented in Section 4). We posit that the failure of existing methods stems from their inability to achieve precise decomposition without paired data. Therefore, our core innovation is an ‘attention-guided decomposition’ framework. We utilize novel modules, including a Feature Preservation Attention Module (FPAM) and a Cascaded Channel-Spatial Attention Module (CSAM), to empower a self-supervised Retinex decomposition. This ensures that crucial enamel textures are preserved while suppressing artifacts under non-uniform light. This approach is further supported by a Random Affine Generation (RAG) module, which forces the network to learn a more robust decomposition by creating challenging image pairs.

In summary, the main contributions of this paper are threefold:

1. We propose a novel hardware system for image acquisition inside water heater tanks, consisting of the IAR robot and IVES software (version 1.0). This system achieves efficient, full-coverage image acquisition within an approximate 50 mm aperture, with an inspection efficiency of 3 min per unit.
2. To the best of our knowledge, we introduce the first public dataset for VE in ESWHs, named the HTIW dataset. It comprises 900 images covering 3 types of non-uniform illumination and 6 representative regions.
3. We propose an effective non-uniform low-light enhancement network, jar-RetinexNet. In a comparison against five state-of-the-art methods, jar-RetinexNet demonstrates superior performance on multiple no-reference metrics and in visual quality.

## 2. Related Works

### 2.1. NUI Enhancement in Industrial Production

Enhancement for NUI in ESWHs is a niche problem. In the broader industrial context, traditional methods like MSRCR [9] and CLAHE [10] are limited by halo artifacts and noise amplification. Deep learning methods initially utilized supervised. training on paired data, with models like URetinexNet [11], SNR-aware [12], and Transformer-based architectures like LLFormer [13], Retinexformer [14], and RetinexMamba [15] achieving strong results. However, as paired data is scarce, zero-reference and unpaired approaches became a research hotspot, including methods like RetinexNet [16], EnlightenGAN [17], Zero-DCE [18], and SCI [19]. The field continues to advance rapidly, incorporating ViT-based attention mechanisms [20], alternative physical models (e.g., dehazing) [21], and powerful generative models, including diffusion-based (DiffDark [22]), VLM-guided (GPP [23]), and text-driven (TSCnet [24]) frameworks. However, these recent SOTA methods, particularly generative, diffusion, and text-driven models [22,23,24], present significant deployment barriers. Their substantial computational overhead is unsuitable for the real-time demands of our IVES system (which requires processing in under 2 min). Furthermore, they are general-purpose enhancers, not specifically tailored to the unique physics of NUI on curved, reflective surfaces for the primary goal of downstream defect detection. Therefore, a gap remains for a robust, efficient, and physically-interpretable algorithm designed for this specific industrial challenge.

### 2.2. Image Acquisition (Systems) for VE in ESWHs

Given the critical importance of the enamel coating for the Visual Examination (VE) of ESWHs, the rapid, comprehensive, and clear acquisition of VE data is essential. The industrial production of ESWHs predominantly relies on manual visual inspection. While manual visual inspection can yield satisfactory results, it suffers from significant limitations, including high labor intensity, subjectivity, and low efficiency, which motivates the development of automated systems [25]. With the advancement of deep learning technologies, automated visual inspection systems have become increasingly prevalent in industrial applications, particularly for surface defect detection [26]. However, the quality of the images captured by these systems often fails to meet the required standards, which severely compromises the accuracy of subsequent processing. More importantly, to the best of our knowledge, no public NUI dataset for VE currently exists, an absence that significantly impedes research into NUI visual enhancement methods. Based on the preceding discussion, this paper aims to systematically address these issues by constructing a comprehensive visual enhancement system for NUI in ESWHs. Furthermore, we contribute the HTIW dataset, built using authentic VE images, and propose an efficient enhancement network, jar-RetinexNet.

## 3. Proposed System and Dataset

### 3.1. Low-Light Image Enhancement System

**Figure 2:**
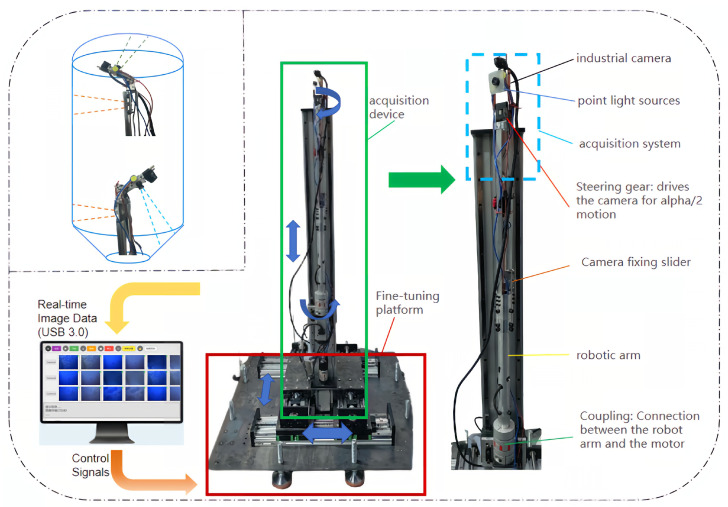
Low-light enhancement system for water heater tank inner walls. Top-left inset shows top/bottom surface acquisition. Right side details the IAR’s mechanical structure, including the acquisition device, fine-tuning platform, camera, and light source. Data flow: IVES sends wired control signals to the IAR’s PLC; the IAR camera transmits raw images (1440 × 1080) via USB 3.0 to IVES, which processes them with jar-RetinexNet and displays the enhanced results. The blue arrows indicate the movement directions. The green arrow points to the detailed components. The yellow arrows represent the data and signal flow.

To address the prevalent issues of image distortion, blur, and strong specular reflections in the imaging of water heater tank inner walls, this paper proposes a non-uniform low-light image enhancement system based on Retinex theory. The overall architecture of this system is illustrated in Figure 2. The system is composed of two core components: three-axis Image Acquisition Robot (IAR) and an Interactive Visualization Enhancement Software (IVES).

The IAR is a precision mechatronic system that integrates three core units. The Image Acquisition Unit is equipped with multiple vision modules, each featuring an M12 industrial lens and a dual-point light source positioned at a 90-degree angle to the camera, ensuring accurate image capture. The Three-Axis Drive Unit, consisting of servo motors, an servo drive, and a PLC, adapts to different tank diameters to achieve full-coverage scanning. Meanwhile, the Horizontal Dual-Axis Fine-Tuning Unit, through several servo motors and a PLC, is responsible for precise alignment with the tank aperture and fine-tuning the focus to ensure accuracy. Figure 2 details the working principle of the IAR and its motion components. In operation, the IAR’s three-axis drive unit performs a composite motion along the Z-axis (longitudinal lifting), a horizontal rotation axis, and a vertical rotation axis to achieve a comprehensive scan of the inner wall. To ensure seamless coverage, we designed a dual-vision-module parallel operation strategy: one module, mounted on the vertical rotation axis, captures images of the tank’s top and bottom, while another module, fixed on the horizontal rotation axis, is dedicated to imaging the side walls. The acquisition paths of these two modules are designed with overlap to guarantee the integrity of the final composite image.

The software system, IVES, is an interactive platform that integrates image enhancement, device control, parameter configuration, and data display. After an operator sets the camera and motion parameters via IVES and initiates the task, the entire process becomes highly automated. The IAR performs image acquisition, data is transmitted in real-time to a server via a USB 3.0 high-speed interface, and the system then automatically invokes the built-in non-uniform low-light enhancement algorithm for processing and saving. Upon task completion, the IAR automatically exits and resets, while IVES stitches and visualizes the enhanced, high-quality images in structural order (top-middle-bottom). This system efficiently provides clear, uniformly illuminated images, offering reliable data support for subsequent quality analysis and defect detection.

### 3.2. Dataset Construction Process

All images in the HTIW dataset were acquired on-site from a water heater enamel production line using our proprietary IAR system. Due to variations in the models and sizes of the water heater tanks on the production line (differing heights and radii), we manually adjusted acquisition parameters and lighting angles via the IVES before each capture session. This process was guided and supervised by a professional inspector specializing in inner-wall image quality to accommodate specific working conditions and ensure the representativeness of the images. After acquisition, we carefully selected 360 typical non-uniform low-light images from the original captures. To genuinely reflect the distribution in real-world scenarios, the vast majority of images were retained, with only a very small number of highly noisy or severely blurred samples being discarded. Finally, to strike a balance between image precision and processing efficiency, the size of all images was standardized to 1440×1080 pixels.

**Figure 3:**
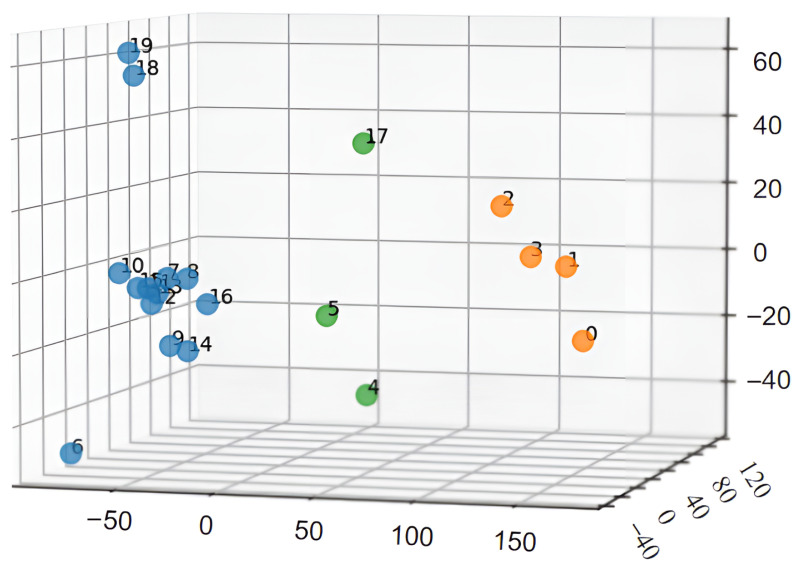
3D visualization of different illumination uniformity categories after PCA. The different colors represent different categories of illumination uniformity as indicated in the legend.

To gain a deeper understanding of the dataset’s characteristics, we performed a quantitative analysis of the images. We extracted “grayscale histogram features” and “block-wise brightness mean features” from each image and concatenated them into a joint feature vector. Subsequently, we applied Principal Component Analysis (PCA) to reduce the dimensionality of this joint vector for 3D visualization. The distribution of the data points after PCA dimensionality reduction is clearly depicted in Figure 3, from which three main clusters of non-uniform illumination can be distinctly observed.

1. Uniform Low-Light Distribution. Primarily found at the top and bottom regions of the tank (indices 0–3).
2. Bright Center, Dark Edges. Mainly appears on the side walls and around small aperture areas of the tank (indices 6–16, 18–19).
3. Dark Center, Bright Edges. Predominantly located in the weld seam areas (indices 4, 5, 17).

**Figure 4:**
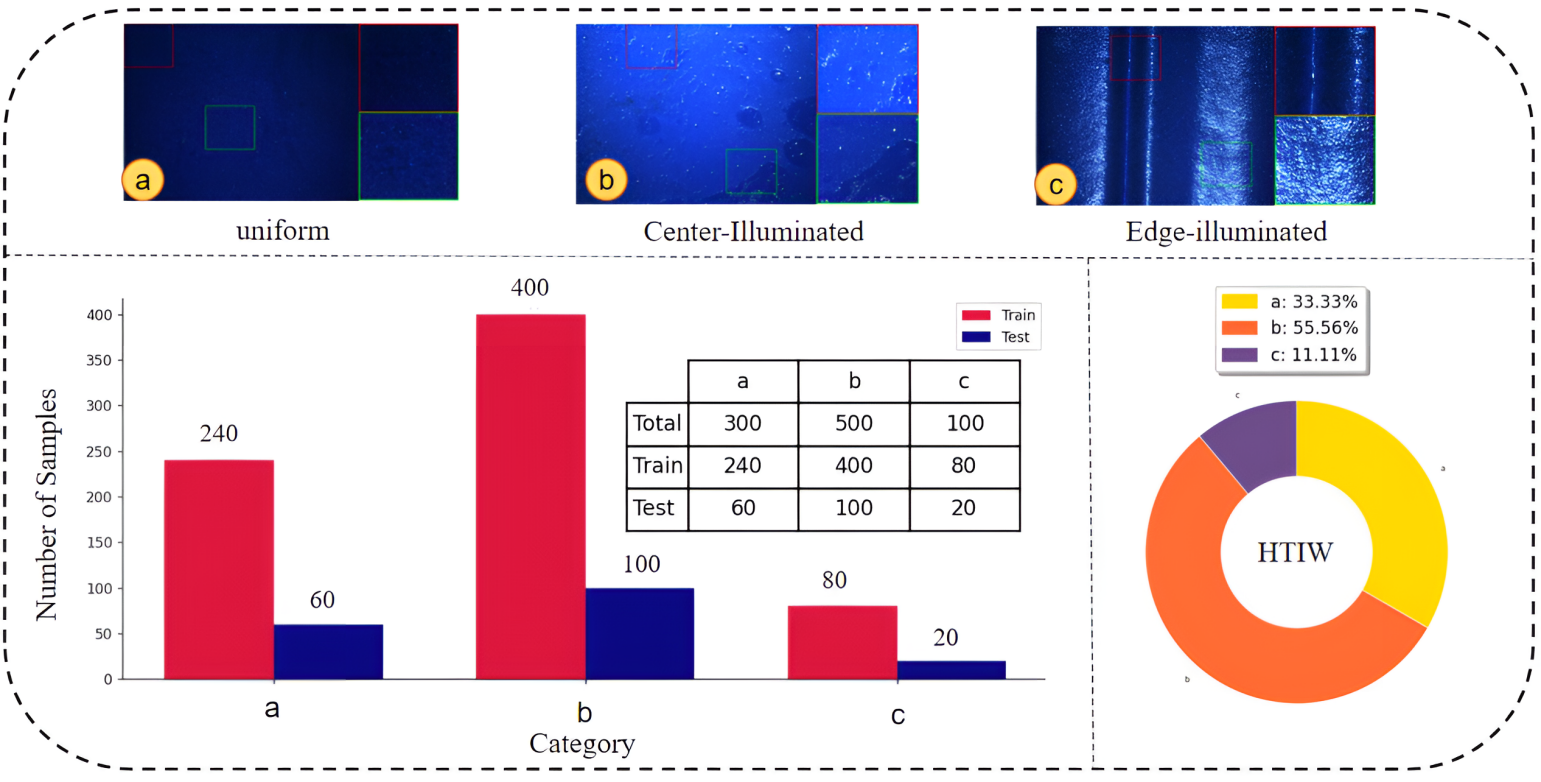
HTIW Dataset distribution (categories a, b, and c represent uniform low-light, center-illuminated, and edge-illuminated images, respectively). The red and green boxes highlight specific regions of interest, with their enlarged views shown in the bottom-right corners of each subfigure.

Following the analysis above, we categorized the 900 images according to the three low-light patterns. To ensure that the distribution of the training and test sets was consistent with real-world production scenarios, we performed random sampling from each category at a ratio of 8:2. This ultimately resulted in the construction of a training set containing 800 images and a test set containing 200 images. The detailed distribution of the dataset is illustrated in Figure 4.

## 4. Proposed Method

### 4.1. Overview

**Figure 5:**
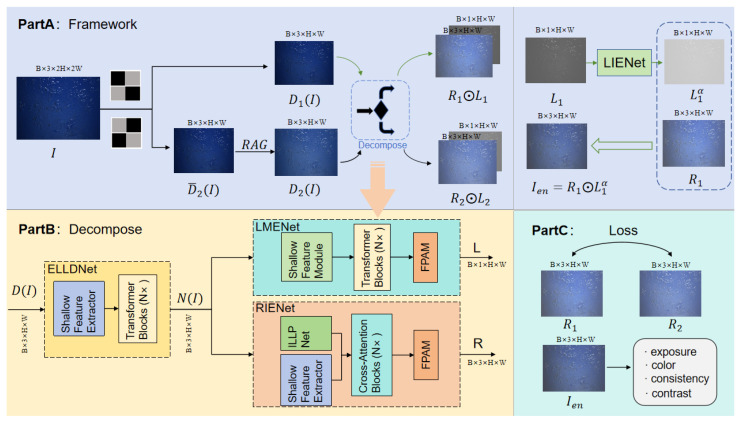
Framework of jar-RetinexNet. The framework illustrates two distinct processes: (1) The Self-Supervised Training Flow: An input image $I\in R^{B\times 3\times 2H\times 2W}$ generates two sub-images, $D_{1}\left(I\right)$ and $D_{2}\left(I\right)$ (via RAG). Both are passed through the Decompose module (Part B) to yield reflectance maps $\left(R_{1},R_{2}\right)$ and illumination maps $\left(L_{1},L_{2}\right)$. (2) The Enhancement/Inference Flow (Green Arrows): This path, which is used during both training and final inference, takes $L_{1}$ and $R_{1}$ as input. $L_{1}$ is fed to LIENet to predict an enhancement coefficient α, producing $L_{en}=L_{1}^{\alpha }$. This is combined with $R_{1}$ to generate the final enhanced image $I_{en}=R_{1}⊙L_{en}$. (Part C) Loss: During training, the self-supervised losses are computed using the outputs from both flows ($R_{1},R_{2}$, and $I_{en}$). All key modules preserve the tensor dimensions, as labeled.

Inspired by the success of Retinex-based deep learning methods [5], we propose a novel model, jar-RetinexNet. Its core architecture is a deep Retinex decomposition model designed to decompose a non-uniform low-light input image *I* into an illumination layer *L* and a reflectance layer *R*. To achieve this in a self-supervised manner, we introduce a specific preprocessing pipeline (including Neighboring Pixel Masking and RAG) to create challenging sub-image pairs. The network is then composed of two main stages: (1) a Decompose module, which uses a novel degradation feature extractor (ELLDNet) to feed two branches (LMENet and RIENet) that separate *L* and *R*, and (2) an Enhancement module (LIENet) that adaptively corrects the estimated illumination. The complete end-to-end architecture is illustrated in Figure 5—and detailed in its caption—while the components are explained in the following sections.

### 4.2. Random Affine Generation Module

To guide the network in effectively decomposing reflectance under a self-supervised learning paradigm., without supervision from paired normal-light images, we introduce the RAG. The core function of this module is to apply a series of non-linear transformations and degradation operations to the input sub-image ${\bar{D}}_{2}\left(I\right)$.

These degradations are not arbitrary; they are physically motivated to proactively simulate the real-world industrial challenges. In the practical working environment, the CMOS sensor operating at high temperatures (70–80 °C) induces significant thermal noise and color drift. Therefore, the “noise addition” and “color jittering” operations within RAG are specifically included to mimic this exact sensor degradation caused by high temperatures. By incorporating these simulated physical degradations (along with other non-linear adjustments, blurring, and Gamma correction), we generate a perturbed image $D_{2}\left(I\right)$. This process is expressed as

(1) $$D_{2}\left(I\right)=\mathrm{RAG}\left({\bar{D}}_{2}\left(I\right)\right)$$

where RAG(·) represents the random affine generation operation. By creating samples under these simulated harsh conditions, the RAG forces the decomposition network to learn a decoupling that is robust against real-world industrial noise, significantly enhancing the model’s generalization capability to complex illumination and sensor degradations.

### 4.3. Decomposition Module

According to Retinex theory [5], an image *I* can be decomposed into a reflectance layer *R* and an illumination layer *L*, related by I=R⊙L, where ⊙ denotes element-wise multiplication. Similarly, low-light images and their enhanced versions can also be decomposed. While traditional Retinex-based networks often employ multi-head and cross-attention mechanisms for this task, we argue these may be insufficient for the complex non-uniform illumination and fine-grained textures in our hot water heater tank images. Therefore, in addition to these mechanisms, our model incorporates channel attention, spatial attention, and multi-scale feature fusion to achieve a more refined separation. The core components of our decomposition module include ELLDNet, LMENet, and RIENet.

#### 4.3.1. ELLDNet

**Figure 6:**
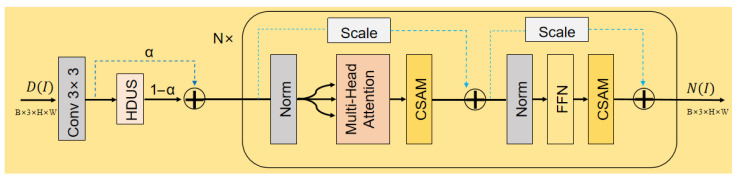
Architecture of the ELLDNet network. An input feature D(I) (dimension $R^{B\times 3\times H\times W}$) is first processed by a Conv3×3 layer to produce an intermediate feature map, $F_{conv}$. This map is then split and fused via a weighted sum: one path, weighted by α, is an identity mapping, while the parallel path, weighted by (1−α), passes through the HDUS module. The resulting fused feature enters *N* cascaded Transformer blocks. Inside each block, the data flows sequentially through Norm → Multi-Head Attention → CSAM and Norm → FFN → CSAM, with two key residual connections (blue dashed arrows) preserving information across these stages. The final output is the degradation feature N(I), which maintains the original input dimension of $R^{B\times 3\times H\times W}$.

As a core feature processing unit in our model (see Figure 6 for its detailed structure), ELLDNet is constructed by stacking *N* identical processing modules. Each module integrates multi-scale feature fusion and attention mechanisms to efficiently extract and recalibrate features.Before features enter the main attention network, we first employ a Hierarchical Down-Up Sampling module (HDUS) that utilizes a two-layer structure to perceive feature changes at different resolutions, which is especially crucial for spatially smooth attributes like illumination. For an input feature map $F_{in}\in R^{H\times W\times C}$, the HDUS operation is

(2) $$F_{\mathrm{out}}=F_{\mathrm{in}}+{\mathrm{Up}}_{2\times }\left({\mathrm{Up}}_{2\times }\left({\mathrm{Down}}_{1/2}\left({\mathrm{Down}}_{1/2}\left(F_{\mathrm{in}}\right)\right)\right)\right)$$

where ${\mathrm{Down}}_{1/2}\left(\cdot \right)$ is a downsampling operation (convolution with stride 2) and ${\mathrm{Up}}_{2\times }\left(\cdot \right)$ is a 2× upsampling operation (bilinear interpolation). This residual connection effectively fuses multi-scale information with minimal computational overhead.

The features enhanced by HDUS are then fed into *N* cascaded core processing modules. Each module contains a Multi-Head Self-Attention (MHSA) layer and a Feed-Forward Network (FFN) layer, with each layer being followed by a CSAM for feature refinement. For an input feature $X_{i}$ to the *i*-th core module, the workflow is as follows:

1. Multi-Head Self-Attention Layer:
  (3) $$X_{i}^{\prime }=\mathrm{CSAM}\left(\mathrm{MHSA}\left(\mathrm{LN}\left(X_{i}\right)\right)\right)+X_{i}$$
  where LN(·) is Layer Normalization. This layer captures long-range dependencies among features.
2. Feed-Forward Network Layer:
  (4) $$X_{i+1}=\mathrm{CSAM}\left(\mathrm{FFN}\left(\mathrm{LN}\left(X_{i}^{\prime }\right)\right)\right)+X_{i}^{\prime }$$
  The FFN layer, consisting of two linear transformations and a non-linear activation, performs point-wise feature transformation.
3. CSAM: To enhance the network’s feature representation, we embed a CSAM after both the MHSA and FFN layers. Unlike standard sequential attention (e.g., CAM then SAM), our industrial images contain both large-scale, low-frequency illumination properties (best captured by channel attention) and fine-grained, high-frequency textures/defects (best captured by spatial attention). A sequential-only approach risks suppressing one feature type while refining the other. Our CSAM therefore adopts a parallel, cascaded structure. The module adaptively recalibrates features by fusing two parallel attention branches. The first branch sequentially applies a Channel Attention Module (CAM) and a Spatial Attention Module (SAM), while the second branch applies only the SAM to the original input. The final output is a weighted sum of these two branches, where the balance is controlled by a learnable parameter α. For an input feature $F_{in}$, the operation is:
  (5) $$F_{\mathrm{out}}=\alpha \cdot \mathrm{SAM}\left(\mathrm{CAM}(F_{\mathrm{in}})\right)+\left(1-\alpha \right)\cdot \mathrm{SAM}\left(F_{\mathrm{in}}\right)$$
  CAM learns and applies weights to different channels, while SAM subsequently learns and applies weights to different spatial locations. This guides the network to focus on information-rich channels and regions, thereby enhancing feature discriminability.

#### 4.3.2. LMENet

**Figure 7:**
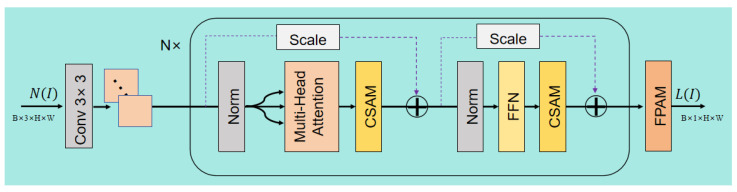
Architecture of the LMENet network. The degradation feature N(I) (from ELLDNet), with an input dimension of $R^{B\times 3\times H\times W}$, is first processed by a Conv 3×3 layer and a shallow feature module. This feature map then enters *N* cascaded Transformer blocks. Within each block, the data flows sequentially through Norm → MHSA → CSAM and Norm → FFN → CSAM, with two crucial residual connections (purple dashed arrows) preserving information across these stages. The resulting feature map is passed to the final FPAM, which refines details and produces the final illumination map L(I). The final output is a single-channel map with dimension $R^{B\times 1\times H\times W}$.

This network (see Figure 7) is specifically designed for illumination layer decomposition. Its main body consists of *N* cascaded instances of the Core Processing Module (identical to the block in ELLDNet, see Equations (2) and (3)). After the features are processed through these core modules, yielding an output $F_{out}$, they are passed through a final FPAM.

The illumination map L(I) must be spatially smooth yet structurally consistent with the scene. Standard attention modules can be overly aggressive, removing structural details during refinement. The FPAM is designed to prevent this by forcing the Spatial Attention Module (SAM) to act as a “refiner” rather than a “generator”. It achieves this via a specific residual connection: the output of the Channel Attention Module (CAM) is added to the original input feature *F* before being fed into the SAM. This ensures that the SAM’s input contains the complete original structure (+F), forcing it to learn only what to emphasize or suppress rather than what to create, thus preserving the critical structural information of the illumination map. Its operation can be defined as

(6) $$\mathrm{FPAM}\left(F\right)=\mathrm{SAM}\left(\mathrm{CAM}(F)+F\right)$$

This module captures rich contextual information to generate the final, detail-accurate illumination map L(I):

(7) $$L\left(I\right)=\mathrm{FPAM}\left(F_{out}\right)$$

Through the final processing by FPAM, the network is able to output an illumination map with richer structural information and spatial details.

#### 4.3.3. RIENet

**Figure 8:**
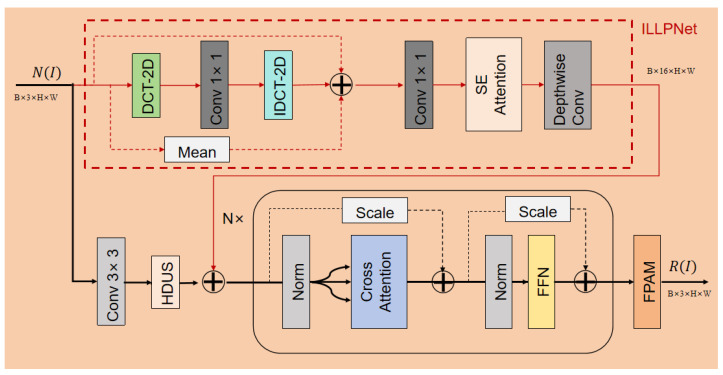
Architecture of the RIENet network, detailing its dual-branch data flow and tensor dimensions. The module takes the degradation feature N(I) (input dimension $R^{B\times 3\times H\times W}$) and processes it via two parallel branches: (1) The top ILLPNet branch (red dashed box, red data flow lines) extracts a multi-channel illumination-prior, $F_{prior}$, with dimension $R^{B\times 16\times H\times W}$. (2) The bottom spatial branch (Conv 3×3, HDUS, black data flow line) extracts a spatial feature, $F_{spatial}$. The core data flow then enters the *N* Transformer blocks, where the Cross-Attention mechanism is guided: the summed feature ($F_{spatial}+F_{prior}$) serves as the Query (Q), while the original $F_{prior}$ (red line) provides the Key (K) and Value (V). Internal residual connections (dashed lines via “Scale” layers) are used within the blocks. Finally, the output is passed through an FPAM to produce the final reflectance map R(I), which is projected back to the dimension $R^{B\times 3\times H\times W}$.

The network decomposes the reflectance layer based on the input feature map and illumination prior. The core challenge of decomposition is to separate the reflectance *R* from the illumination *L*. In the feature space, this can be modeled as “subtracting” the illumination information from the mixed features to isolate the reflectance. RIENet achieves this decoupling using Cross-Attention. It first generates two distinct feature streams from the input N(I): (1) a spatial feature stream ($F_{spatial}$) via a Conv 3×3 and HDUS branch, which represents the mixed scene content (analogous to *I*), and (2) an illumination-prior stream ($F_{prior}$) via the lightweight ILLPNet (red dashed box), which represents the lighting conditions (analogous to *L*). These streams are then fed into the $N_{x}$ Transformer blocks. The Cross-Attention module (Figure 8) within these blocks is configured to perform the crucial decoupling:

1. Query (Q): The fused feature $F_{spatial}+F_{prior}$ is used as the Query. This asks the following question: “For this mixed feature, what illumination information does it contain?”
2. Key (K) & Value (V): The illumination-prior $F_{prior}$ is used as both the Key and Value. This provides the following answer: “This is the illumination information you should look for and subtract.”

This mechanism forces the network to attend to and remove the illumination-specific features ($F_{prior}$) from the mixed spatial features, thereby isolating the illumination-invariant reflectance R(I). The output is finally passed through an FPAM to refine the reflectance map details.

(8) $$\begin{array}{cc} R(I)=\mathrm{FPAM}(\mathrm{CrossAttention} & \left(\mathrm{HDUS}(\mathrm{Conv}3\times 3(N(I)))\right)\\ & +\mathrm{ILLPNet}(N(I))) \end{array}$$

where D(I) is the feature map of the input image and Conv 3×3 is a 3×3 convolutional layer. The CrossAttention module fuses the outputs from the Conv 3×3 layer and the previously introduced HDUS. Finally, these fused features are processed by the aforementioned FPAM module to produce the final reflectance layer R(I).

#### 4.3.4. LIENet

This network receives the decomposed illumination layer $L_{1}$ as input. After processing its features, it applies Global Average Pooling. The pooled features are scaled through two linear layers into a one-dimensional enhancement factor α, used to adaptively correct the illumination map. This enhancement factor is ultimately used to adjust $L_{1}$ to generate the enhanced illumination layer $L_{en}$.

### 4.4. Loss Function

For model training, we adopt the multi-component composite loss function proposed by Li et al. [27], which has proven effective for joint denoising and low-light enhancement tasks. The total loss, $L_{total}$, is composed of a Retinex decomposition loss and a self-supervised enhancement loss.

#### 4.4.1. Retinex Decomposition Loss

This component ensures the network accurately decomposes the image according to the physical assumptions of Retinex theory. It consists of the following:

1. Reflectance Consistency Loss ($L_{R}$): This term constrains the reflectance maps ($R_{1}$, $R_{2}$) recovered from two sub-images with different degradations to be consistent, based on the prior that an object’s reflectance is illumination-invariant. It is defined as
  (9) $$L_{R}={∥R_{1}-R_{2}∥}_{1}+\omega_{reg}L_{reg}$$
  where $L_{reg}$ is a cross-scale regularization term to enhance generalization and $\omega_{reg}$ is the regularization coefficient.
2. Illumination and Reconstruction Loss ($L_{L}$): This term comprises a reconstruction loss to ensure fidelity and a smoothness loss to enforce the gradual nature of natural illumination. It is formulated as
  (10) $$L_{L}=L_{recon}+\lambda_{smooth}L_{smooth}$$

#### 4.4.2. Self-Supervised Enhancement Loss

This component guides the enhanced image, $I_{en}$, towards better perceptual quality without a reference image. It includes the following:

1. Spatial Consistency Loss ($L_{con}$): This loss preserves the image’s structure and content by maintaining the consistency of gradients between the image before and after enhancement.
2. Enhancement Effect Loss ($L_{enh}$): This loss balances two aspects of enhancement: an exposure loss ($L_{exp}$) that drives local brightness towards a well-exposed level, and a color constancy loss ($L_{color}$) to prevent color casts. Its formula is
  (11) $$L_{enh}=L_{exp}+\lambda_{color}L_{color}$$

Finally, our total training objective is the weighted sum of these components:

(12) $$L_{total}=\omega_{R}L_{R}+\omega_{L}L_{L}+\omega_{con}L_{con}+\omega_{enh}L_{enh}$$

where $\omega_{R},\omega_{L},\omega_{con},$ and $\omega_{enh}$ are hyperparameters that balance each loss term.

## 5. Experiments

### 5.1. Implementation Details

Our experiments were conducted on the HTIW dataset. To ensure a fair comparison, all experiments were performed within a unified environment consisting of Ubuntu 24.04, Python 3.9, and the PyTorch framework (version 2.6.0). The hardware utilized was a single NVIDIA GeForce RTX 4090 GPU (NVIDIA Corporation, Santa Clara, CA, USA) with 24 GB of VRAM. We employed the Adam optimizer to update the network parameters. Following standard practice, we configured its hyperparameters to ensure stable and efficient training: the initial learning rate was set to $5\times 10^{-6}$, and the betas were set to (0.9, 0.999). The network was trained for 100 epochs. During the training phase, images were randomly cropped into 256×256 patches, and their pixel values were normalized to the range (0,1). The batch size was set to 1. Under these settings, the jar-RetinexNet model required approximately 6 h of training time.

### 5.2. Evaluation Metrics

Given that the HTIW dataset does not include corresponding ground-truth images, our evaluation relies on two complementary no-reference image quality assessment (NR-IQA) metrics. The selection of these metrics is specifically justified by the goals of industrial visual inspection:

1. Artifact and Distortion Suppression: The primary metric is BRISQUE (Blind/Referenceless Image Spatial Quality Evaluator) [16]. In an industrial context, an enhancement algorithm must not introduce new distortions (e.g., noise amplification, unnatural edges, or blur) that could be mistaken for defects or could obscure them. BRISQUE excels at quantifying such artifacts by evaluating natural scene statistics. A lower score indicates a cleaner, higher-quality image with fewer distortions.
2. Structural Clarity and Naturalness: The second metric is CLIPIQA [28]. While BRISQUE assesses low-level artifacts, CLIPIQA, which is based on a large-scale vision-language model (CLIP), evaluates the overall semantic quality and naturalness. For our task, a “good” enhancement must restore the structural clarity and texture of the enamel surface, making potential defects easily discernible. A low CLIPIQA score signifies that the enhanced image is not only bright but also semantically coherent and structurally clear, which is crucial for subsequent human or automated inspection.

Together, these metrics provide a comprehensive evaluation relevant to our industrial goal: BRISQUE penalizes artifacts, while CLIPIQA rewards structural clarity. A lower score is better for both.

### 5.3. Comparative Experiments

In this study, we compare our proposed jar-RetinexNet against five representative methods: ZERO-DCE [18], CLIP-LIT [29], Sci-medium [19], RUAS [30], and LightDiffusion [31]. Unlike methods such as ZERO-DCE and Sci-medium, our network architecture introduces novel modules to jointly model illumination degradation and noise interference. Specifically, we significantly improve the separation quality of the reflectance and illumination layers through a frequency-guided mechanism. Additionally, we designed a neighborhood mask strategy to achieve self-supervised modeling and removal of real-world noise, enabling the network to possess excellent denoising capabilities even without reference images. Compared to LightDiffusion [31], which progressively generates enhanced images using a diffusion model, our method not only has stronger physical interpretability but also avoids the high computational complexity and slow inference speed associated with generative modeling, making it more suitable for real-time industrial applications. Overall, jar-RetinexNet achieves a superior balance in preserving image structure, restoring color, and enhancing details, allowing it to more effectively address the complex degradations found in real-world industrial scenes.

#### 5.3.1. Quantitative Results

**Figure 9:**
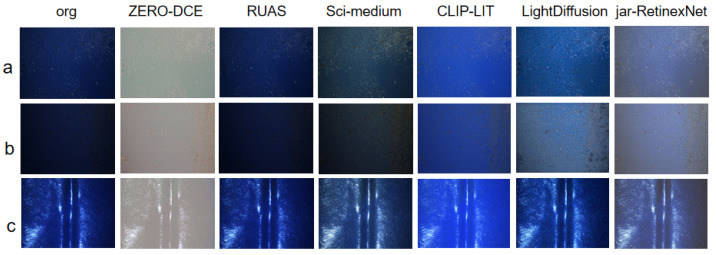
Visual comparison of the proposed method with five state-of-the-art methods on three different low-light scenarios from the HTIW dataset: (**a**) Uniform low-light; (**b**) Center-illuminated; (**c**) Edge-illuminated.

To evaluate the practical applicability of jar-RetinexNet for deployment in industrial systems, we provide a comprehensive quantitative comparison in Table 1, evaluating both the final enhancement quality and the model efficiency required to achieve it. In terms of visual quality, our method achieves the best performance (marked in red). Notably, it scores an average of 25.4457 on BRISQUE ↓ and 0.3160 on CLIPIQA ↓. Furthermore, repeated testing across the dataset confirmed the high stability of our model: the BRISQUE scores consistently fluctuated only within a narrow ±0.90 range (Std. Dev.), and the CLIPIQA scores fluctuated within ±0.04. This stable and superior result demonstrates our approach’s capability in enhancing image naturalness and preserving structural fidelity. The second-best method for quality, ZERO-DCE (marked in blue), also performs well but is slightly behind. In terms of model efficiency, a clear trade-off is observed. Lightweight methods like ZERO-DCE and RUAS have the lowest computational cost (e.g., 1.5 GFLOPs and 0.67 ms for ZERO-DCE). However, as demonstrated in our qualitative results (Figure 9), their enhancement performance on our challenging HTIW dataset is insufficient. Conversely, generative models like LightDiffusion, while visually competitive, are computationally prohibitive. With 25.41 M parameters and 2800.77 GFLOPs, they are unsuitable for the real-time processing demands of our IVES system. Our proposed jar-RetinexNet (Ours) achieves the best balance: while it remains extremely lightweight in parameters (0.31 M), its complex attention-based architecture requires a higher computational load (968.19 GFLOPs) and inference time (216.59 ms). This places it as an optimal trade-off, demonstrating its suitability for deployment in industrial scenarios where high-fidelity, stable enhancement is prioritized over raw inference speed.

#### 5.3.2. Qualitative Results

Figure 9 visually presents the enhancement results of each method on the three different low-light patterns from the HTIW dataset. The HTIW images, captured in an environment with extremely weak and highly non-uniform point light sources, combined with significant variations in surface curvature, effectively amplify the performance differences among algorithms when dealing with complex illumination and surface reflections. As shown in Figure 9a, the competing methods all exhibit noticeable flaws: ZERO-DCE suffers from severe color deviation; Sci-medium and RUAS provide insufficient brightness enhancement; Clip-LIT leads to significant blurring of details; and while LightDiffusion avoids severe artifacts, it still produces uneven exposure and loss of structural details in some regions. In contrast, our proposed jar-RetinexNet not only achieves a more natural and balanced overall brightness enhancement but also demonstrates significant advantages in detail preservation and noise suppression. This superior performance is also validated in other scenes, as shown in Figure 9b,c, confirming that our method obtains the best overall visual quality.

### 5.4. Ablation Study

To verify the effectiveness of the proposed RAG, FPAM, and CSAM attention mechanism in jar-RetinexNet, we conducted multiple ablation experiments. The relevant quantitative results are shown in Table 2. In this table, we also use red and blue to denote the best and second-best performance, respectively.

1. Importance of RAG: When the RAG is removed (i.e., the row indicated by × RAG), the model’s performance on both BRISQUE and CLIPIQA no-reference metrics decreases. This indicates that the RAG is crucial in guiding the model to learn natural, smooth, and consistent illumination distributions, enhancing the network’s adaptability and generalization performance to non-uniform illumination scenes.
2. Effectiveness of FPAM and CSAM Attention Mechanisms: As can be seen from Table 2, Whether increasing FPAM, CSAM, or both simultaneously, the performance of the BRISQUE and CLIPIQA metrics increases significantly, indicating a marked improvement in image quality. This strongly demonstrates that the FPAM and CSAM attention mechanisms play an important role in improving the structural fidelity and perceptual quality of enhanced images, effectively promoting the extraction and representation of useful features in images, especially crucial for the precise separation of reflectance and illumination layers.

These ablation experiment results clearly prove the rationality and effectiveness of our proposed module designs, which work collaboratively to significantly improve the overall performance of jar-RetinexNet.

### 5.5. Hyperparameter Sensitivity Analysis

To provide a deeper analysis of our model, we justify our chosen hyperparameters. For the loss weights in Equation (12) ($\omega_{R}=1.0,\omega_{L}=0.5$, etc.), we adopted the settings directly from the validated work of Li et al. [27]. This approach ensures our network is guided by a proven physical and perceptual prior for self-supervised decomposition. We then analyze the “intensity” of our novel RAG module (Section 4.2), as this is a core contribution. To isolate its impact, this analysis was performed on the baseline model (i.e., without FPAM/CSAM).

We tested four levels: ‘None’ (RAG removed), ‘High’ (extreme blur + high noise), ‘Low’ (color jitter + gamma), and ‘Medium’ (our proposed setting: Low + blur + noise). As shown in Table 3 (best results in red, second-best in blue), the ‘None’ (baseline) model performs poorly. ‘Low’ intensity is insufficient, while ‘High’ intensity degrades performance, likely by decorrelating the sub-images ($D_{1},D_{2}$) and violating the reflectance consistency assumption. Our ‘Medium’ (Proposed) setting provides the optimal balance, achieving the best scores in this baseline comparison (CLIPIQA 0.3460) and forcing the network to generalize effectively.

### 5.6. Downstream Task Validation: Defect Detection

The ultimate goal of our system is to provide high-quality visual data for industrial quality control, specifically automated defect detection. To validate jar-RetinexNet’s practical utility in this downstream task, we conducted a preliminary experiment. We manually labeled 150 images from our enhanced HTIW test set, identifying common defects such as “dents” and “blackening”. We then trained a standard lightweight object detector, YOLOv5-s, on two different datasets: (1) the original low-light images, and (2) the corresponding images enhanced by our jar-RetinexNet. Both models were trained for 50 epochs under identical settings. As shown in Table 4, the results are definitive. The detector trained on original images struggled significantly, achieving a mean Average Precision (mAP@0.5) of only 45.2%. In contrast, the detector trained on our enhanced images achieved a mAP@0.5 of 72.8%. This 27.6% improvement in mAP demonstrates that jar-RetinexNet effectively enhances critical defect features, providing a more stable and feature-rich input that significantly boosts the performance of automated inspection algorithms.

### 5.7. Practical Application

Our proposed image enhancement system is designed to achieve rapid, full-coverage, and automated enhancement of the inner walls of water heater tanks, which have an approximate 50mm aperture. Crucially, our proprietary Interactive Visualization Enhancement Software (IVES) not only presents high-clarity images to inspectors for intuitive assessment but also stores these high-quality images on a Network Attached Storage (NAS) system. This provides a reliable data foundation for subsequent automated defect detection algorithms. Extensive testing in a real-world factory environment has demonstrated that the system fully meets the demands of the industrial production pace. Specifically, for a standard 700 mm-high ESWH, the Image Acquisition Robot (IAR) completes a full-coverage scan in just 11 rotations. With each rotation taking 10 s, the total acquisition time is 110 s. During this process, five vision units capture approximately 313 images, which are transferred in real-time to the processing unit via USB 3.0. Thanks to the system’s parallel design, our Jar-RetinexNet enhancement algorithm processes the images concurrently on an NVIDIA RTX 4090 GPU in approximately 100 s. Factoring in a 10-s equipment reset time, the total cycle time to process a single water heater tank is 120 s. Thus, the system’s average processing time per inner tank is two minutes, significantly surpassing the industrial standard of one unit per three minutes (180 s) and amply demonstrating the system’s high efficiency and precision in practical industrial applications.

### 5.8. Limitations Analysis and Further Discussion

Although jar-RetinexNet demonstrates robust performance, it has identified limitations that guide our future work. A primary limitation is the handling of partial specular reflections. While our IAR hardware is designed to suppress reflections, strong highlights can still occur on highly curved surfaces. As our Retinex-based algorithm was not explicitly designed to remove these, they can occasionally cause visual artifacts. A more explicit mechanism for handling specularities is therefore a key area for future improvement.

A second challenge is adaptability to extreme temperatures (70–80 °C), which induce sensor noise. We proactively addressed this at the algorithmic level: our RAG module (Section 4.2) was physically motivated to simulate this thermal noise. By training on these conditions, jar-RetinexNet learned robustness to such degradations, a design choice quantitatively supported by our hyperparameter analysis (Section 5.5). While future work can investigate hardware-level thermal compensation, our algorithm already provides a validated baseline solution.

Finally, our ultimate goal is full automation. The current system provides high-quality images for manual inspection, laying the data foundation for this next step. Our explicit future work is to develop and integrate an efficient automated defect detection module, establishing a complete “Image Acquisition → Intelligent Enhancement → Automated Detection” pipeline and elevating the system’s applied value.

## 6. Conclusions

In this paper, we have constructed an integrated enhancement system for Non-Uniform Illumination (NUI) in Electric Storage Water Heater (ESWH) scenarios, comprising an Image Acquisition Robot (IAR) and Interactive Visualization Enhancement Software (IVES). This system achieves accurate and efficient Visual Examination (VE) of inner tanks. A key contribution is the creation of the first non-uniform low-light dataset for this application, HTIW, which fills a critical data gap. Most importantly, we proposed the jar-RetinexNet, an attention-guided, self-supervised Retinex network. It effectively addresses the complex challenges of the HTIW dataset, achieving state-of-the-art enhancement performance and providing a robust data source for optimizing the industrial production workflow.

Looking ahead, our work will focus on two specific and critical directions for industrial deployment. First, we will move beyond enhancement to create a fully integrated, closed-loop intelligent inspection system. This primary goal involves developing a dedicated defect detection module (e.g., for “dents” and “blackening”) that is seamlessly integrated with the current IVES. This will transform our system from a visual aid into a fully automated “acquisition-enhancement-detection” pipeline, realizing true smart manufacturing. Second, we will address the challenge of extreme industrial environments, particularly the high-temperature conditions (70–80 °C) encountered during production. This future work will involve investigating both hardware-level adaptations, such as thermal compensation for the imaging sensors, ensuring system stability and reliability.

## Figures and Tables

**Table 1:** Quantitative comparison on the HTIW dataset, evaluating model complexity (Params, FLOPs, AIT) and visual quality (BRISQUE ↓, CLIPIQA ↓). FLOPs and AIT are measured on a 1440×1080 image. The best results are highlighted in red, and the second-best results are in blue.

Method	Params (M)	FLOPs (G)	AIT (ms)	BRISQUE ↓	CLIPIQA ↓
RUAS	0.01	18.2	4.87	32.8689	0.3609
ZERO-DCE	0.08	1.5	0.67	26.4096	0.3293
Sci-medium	0.01	271.98	6.63	29.2346	0.3839
Clip-LIT	0.27	432.92	111.49	34.0603	0.4393
LightDiffusion	25.41	2800.77	372.25	42.2949	0.4115
Ours	0.31	968.192	216.59	25.4457 ± 0.90	0.3160 ± 0.04

**Table 2:** Ablation study on the contributions of RAG, FPAM, and CSAM on the HTIW dataset, using BRISQUE ↓ and CLIPIQA ↓ metrics. The best and second-best results are highlighted in red and blue, respectively. The symbols ‘✓’ and ‘×’ indicate the inclusion and exclusion of the corresponding module, respectively.

RAG	FPAM	CSAM	BRISQUE ↓	CLIPIQA ↓
×	×	×	39.4295	0.4299
×	✓	×	39.2021	0.3645
✓	✓	×	26.6651	0.3268
×	✓	✓	25.4706	0.3260
			25.4457	0.3160

**Table 3:** Sensitivity analysis of RAG perturbation intensity on the HTIW dataset. These tests were performed on the baseline model (without FPAM/CSAM) to isolate the effect of RAG. The best and second-best results are highlighted in red and blue, respectively. The downward arrow (↓) indicates that lower values represent better performance.

RAG Intensity	Description	BRISQUE ↓	CLIPIQA ↓
None	RAG module removed	39.4295	0.4299
High	Extreme Blur + High Noise	40.9128	0.4358
Low	Color Jitter + Gamma	35.1543	0.3781
Medium (Ours)	Low + Blur + Noise	31.4362	0.3460

**Table 4:** Downstream defect detection performance (mAP@0.5) using a YOLOv5-s detector.

Training Data	mAP@0.5 (%)
Original Low-Light Images	45.2
jar-RetinexNet Enhanced (Ours)	72.8

## Data Availability

The data that support the findings will be available in [HTIW] at [https://github.com/tiankongkongkongruye/HVAB] (accessed on 18 November 2025) following an embargo from the date of publication to allow for commercialization of research findings.
